# From Population Averaging to Single Event Resolution: Evolution of Sensing Platforms for Membrane Fusion

**DOI:** 10.3390/s26051669

**Published:** 2026-03-06

**Authors:** Yazhuo Feng, Xuanzhu Zhao, Zhangbao Sun, Zhangrong Lou, Sheng Zhang

**Affiliations:** 1 Faculty of Medicine, Dalian University of Technology, Dalian 116024, China; fengyazhuo@mail.dlut.edu.cn; 2 College of Medical Laboratory Science, Dalian Medical University, Dalian 116044, China; 72109019@mail.dlut.edu.cn; 3 School of Future Technology, Dalian University of Technology, Dalian 116024, China; 2972944250@mail.dlut.edu.cn; 4 State Key Laboratory of Fine Chemicals, Frontier Science Center for Smart Materials, School of Chemical Engineering, Dalian University of Technology, Dalian 116024, China

**Keywords:** membrane fusion, sensors, fluorescence resonance energy transfer, black lipid membranes, solid supported lipid bilayers, electrochemical impedance spectroscopy

## Abstract

Membrane fusion is fundamental to intracellular transport and signal transduction, with its dysregulation implicated in various diseases. Deciphering its transient, microscale dynamics requires advanced sensing technologies. This review systematically evaluates optical and electrochemical sensing platforms for in vitro studies of membrane fusion. Optical sensing platforms provide greater intuitive readout of membrane fusion events, whereas electrochemical sensing platforms enable label-free, single-event resolution. We revisit classical fluorescence resonance energy transfer (FRET) strategies for lipid and content mixing, tracing their evolution from ensemble measurements to real-time, multiparameter, single-vesicle analysis. We further examine electrochemical platforms based on nanodisc-black lipid membranes (ND-BLMs) and solid-supported lipid bilayers (SLBs), highlighting their unique capabilities in characterizing fusion pore kinetics and virus–host membrane fusion. ND-BLM-based systems are irreplaceable for probing fusion pore kinetics, owing to their sub-millisecond temporal resolution and being essentially free from ion saturation and depletion effects. Meanwhile, SLB-based electrochemical sensing platforms excel at high-throughput detection of viral membrane fusion events by virtue of their excellent compatibility and facile integration. These sensors provide powerful tools for elucidating the molecular mechanisms underlying SNARE-mediated membrane fusion and viral fusion processes. Finally, this review outlines future directions centered on the integration of multimodal sensing and the construction of physiomimetic membranes, emphasizing the critical role of cross-scale, multiparameter sensing in bridging molecular mechanisms with biological functions and advancing the diagnosis and treatment of membrane fusion-related diseases.

## 1. Introduction

Biological membranes are the fundamental structural and functional core of all living organisms. In aqueous environments, lipid molecules spontaneously assemble into bilayer structures driven by their amphiphilic nature. The resulting hydrophobic core makes the membrane naturally impermeable to hydrophilic molecules, creating a physical barrier that separates the intracellular environment from the external surroundings, ensuring the spatiotemporal organisation of complex biological processes [1,2,3,4]. As the critical process involving the merger of lipid bilayers and the exchange of internal content, membrane fusion serves as the central mechanism for material transport and signal transduction. It is fundamentally involved in vital activities such as intracellular trafficking, neurotransmitter release, immune responses, and viral entry, playing a decisive role in maintaining cellular homeostasis [5,6,7,8,9]. Defects in these fusion mechanisms frequently underlie the development of various diseases [10,11,12,13,14,15]. Given the inherent complexity of in vivo environments, in vitro reconstitution systems have emerged as ideal platforms for revealing the fundamental mechanisms of fusion. This article focuses on the use of sensing technologies to detect two key fusion events: vesicle–plasma membrane fusion and the entry of enveloped viruses into host cells. High-performance sensing technologies represent essential tools for resolving these microscopic fusion events and elucidating the underlying mechanisms.

### 1.1. Vesicle-Plasma Membrane Fusion

**Figure 1:**
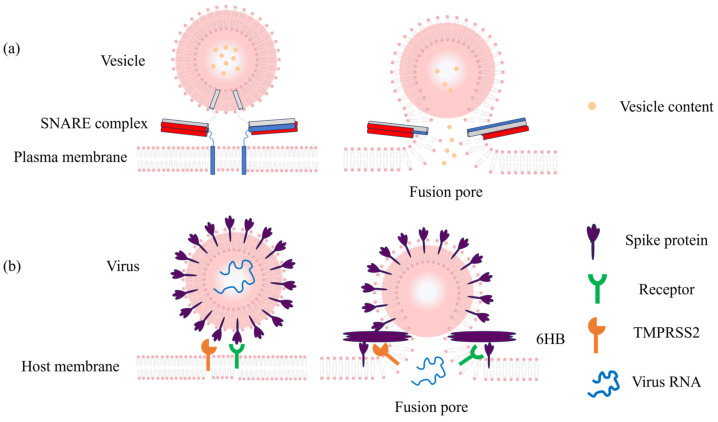
Schematic illustrations of vesicle-plasma membrane fusion and virus–host membrane fusion. (**a**) Vesicle-plasma membrane fusion: Prior to fusion, the v-SNARE (VAMP2, gray) on the vesicle and the t-SNAREs (Syntaxin, blue and SNAP25, red) on the plasma membrane bind to form a SNARE complex. The SNARE complex acts like a zipper, bringing the vesicle closer to the plasma membrane. Ultimately, the vesicle membrane fuses with the plasma membrane, forming a fusion pore through which the vesicle contents are released. (**b**) Receptor-mediated virus–host cell membrane fusion: The viral fusion protein (spike protein, purple) on the viral envelope binds to the host cell receptor (green) and is subsequently cleaved by TMPRSS2, a serine protease anchored in the host cell membrane. The fusion protein then undergoes a conformational rearrangement, exposing the fusion peptide. This peptide inserts into the host cell membrane, and the fusion protein further folds to form a six-helix bundle (6HB) structure, bringing the viral envelope closer to the host cell membrane. Finally, the viral envelope fuses with the host membrane, forming a fusion pore through which the viral genetic material is released. Note: image not drawn to scale.

In eukaryotes, the specific fusion of vesicles with the plasma membrane is controlled by the evolutionarily conserved SNARE (soluble N-ethylmaleimide-sensitive factor attachment protein receptor) protein family. During synaptic neurotransmission, SNARE complexes drive the precise and controlled fusion of synaptic vesicles with the presynaptic membrane. This process progresses through a series of complex intermediate states, including docking, stalk formation, hemifusion, and the opening and expansion of the fusion pore (Figure 1a) [5,7,16,17]. Molecular abnormalities in this pathway constitute a primary pathogenic mechanism for various synaptic disorders [10,11,12,13,14,15]. In Parkinson’s disease, α-Synuclein also interferes with SNARE assembly, hindering pore expansion and altering neurotransmitter release [10,11]. Pathogenic variants in SNAREs and associated proteins also lead to various epileptic disorders [5,12,13]. Similarly, the pathological mechanisms of depression are firmly linked to defects in synaptic vesicle fusion and associated abnormalities [14,15]. Traditional characterisation methods are often inadequate for capturing these transient, microscopic dynamic processes. Specific sensing technologies, however, can convert fusion-related molecular events into quantifiable signals. By quantifying fusion pore expansion kinetics and characterizing how pathogenic proteins regulate vesicle fusion efficiency, these approaches provide direct experimental evidence for the pathological mechanisms of diseases caused by membrane fusion defects stemming from abnormal protein function, which is highly beneficial for understanding the underlying mechanisms. These advancements range from the real-time tracking of lipid mixing kinetics via fluorescence resonance energy transfer (FRET) sensors in batch vesicles, to the detection of fusion pore “flickering” at the single-vesicle level using total internal reflection fluorescence (TIRF) microscopy [14,18,19,20,21,22,23,24,25,26]. And the nanodisc-black lipid membrane (ND-BLM) system further facilitates the conduct of precision-controlled research at the single-molecule level [27,28,29,30]. Sensing technologies have transformed membrane fusion from a “black box” into an observable process, providing useful tools for deciphering SNARE complex assembly rules and the details of fusion pore dynamics.

### 1.2. Virus–Host Membrane Fusion

Like vesicle fusion, the entry of enveloped viruses into host cells depends on fusion proteins on the virus surface. These proteins change their shape when they find a cell receptor or sense changes in environmental pH. This action creates a fusion pore between the virus and the host cell membrane, allowing the virus to pass its genetic material into the host [31,32]. High-sensitivity sensors are now necessary for tasks like quick virus detection and identifying different virus strains (Figure 1b) [33,34,35].

Traditional detection methods usually focus on the final results after an infection has already happened. These endpoint methods cannot track the fusion process in real time. New sensing platforms have solved this problem. Sensors based on solid-supported lipid bilayers (SLBs) and electrochemical impedance spectroscopy (EIS) can measure virus binding and fusion [36,37,38,39,40]. These platforms detect membrane fusion by monitoring changes in membrane resistance.

Organic electrochemical transistor (OECT) sensors are another powerful tool. They use the amplification effect to turn small ion movements into large electrical signals. This makes them very sensitive to fusion events. These technologies allow researchers to track virus fusion as it happens. They also offer a rapid means to evaluate the efficacy of new antiviral drugs [41,42].

The short-lived, microscopic, and complex nature of membrane fusion means that understanding its mechanisms depends heavily on new sensing technologies. Progress in these technologies has driven deeper research into how membranes fuse. This paper reviews optical and electrochemical sensing methods used in in vitro studies. We describe the principles, features, limitations, and optimization strategies of these techniques, as well as their practical applications.

## 2. Principles and Applications of Optical Sensors

### 2.1. Lipid Mixing-Based Detection of Membrane Fusion

To investigate the kinetics and molecular mechanisms of SNARE-mediated membrane fusion, researchers developed an in vitro system using protein-reconstituted vesicles. Since 1998, FRET sensing based on population vesicles has emerged as a key strategy in this field and has evolved into a standard method for membrane fusion research [18].

**Figure 2:**
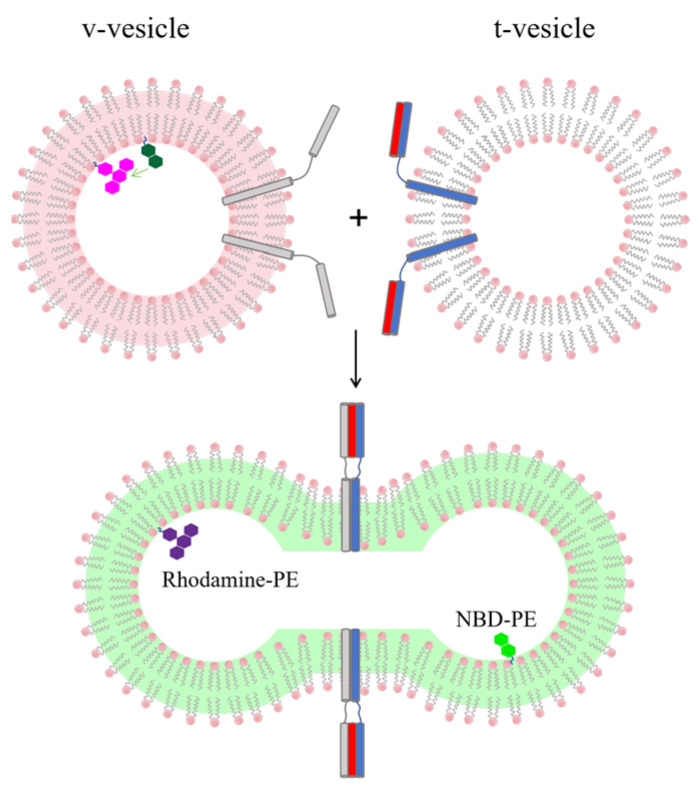
Schematic illustration of the lipid mixing sensor used to monitor membrane fusion. Initially quenched NBD-PE (green) fluoresces upon membrane fusion as the increased distance from Rhodamine-PE (magenta) reduces FRET efficiency, shifting the membrane fluorescence from red to green. SNARE proteins are shown as rod-like structures on the membranes: blue and red represent t-SNAREs, and grey represents v-SNARE. Note: image not drawn to scale.

In this system, v-SNARE (VAMP2) and t-SNARE proteins are separately reconstituted into two different groups of vesicles (v-vesicle and t-vesicle). The first group contains two membrane-anchored fluorescent probes: NBD-PE (donor) and Rhodamine-PE (acceptor). The second group is non-fluorescent and acts as the fusion target. In the initial state, the donor and acceptor probes are closely positioned on the vesicle membrane, resulting in high FRET efficiency. This results in a strong signal from the acceptor and a weak signal from the donor. When SNAREs drive fusion between the two vesicles, the fluorescent molecules spread out and become diluted in the larger lipid bilayer. This distance increase reduces FRET efficiency and significantly boosts the donor’s fluorescence. By constantly tracking the change in the ratio of donor to acceptor intensity (*FRET = I_acceptor_/(I_acceptor_ + I_donor_*)), this sensor provides real-time, quantitative monitoring of lipid mixing (Figure 2) [18].

This sensing method offers several critical advantages. The signal comes directly from the lipid mixing process and is not easily affected by differences in vesicle size. It is especially useful for small vesicles with diameters under 70 nm, where traditional tools like Nanoparticle Tracking Analysis (NTA) often lack sufficient sensitivity. The practical utility of this method is demonstrated across various research focuses. In one study, Yang et al. used NBD-labeled vesicles to reveal a strong size-dependency in fusion, noting that 40–50 nm vesicles fuse up to six times faster than other sizes [19]. Li et al. employed the same sensor to demonstrate that SNARE-mediated tubular protrusions in vesicles suppress the fusion process, thereby offering an additional significant pathway for investigating the pathogenic mechanisms of Parkinson’s disease [20]. In recent research, Zhao et al. innovatively employed NBD dyes to label t-SNARE-containing nanodiscs, enabling the detection of membrane fusion between synaptic vesicles and nanodiscs [14]. They observed that in mice exhibiting depression-like behaviors induced by chronic variable stress, the fusion efficiency of synaptic vesicles was significantly impaired [14].

Beyond the classic NBD/Rhodamine probe pair, researchers have introduced other lipid dye combinations to expand the scope of this sensing platform. Prominent examples include Marina Blue-PE/NBD-PE and the DiO/DiI dye pair, both of which enable direct, real-time monitoring of lipid mixing in population vesicle assays [21,22]. Performance trade-offs exist within these dye combinations. While the Marina Blue-PE/NBD-PE pair offers stronger spectral overlap and FRET efficiency, its shorter excitation wavelength poses phototoxicity risks that limit live-cell studies [23]. The DiO/DiI pair, in contrast, favors live-cell imaging through longer excitation wavelengths and reduced cellular damage, albeit with lower FRET efficiency [24]. Moreover, the differential diffusion rates of DiO and DiI within membranes can result in less stable FRET signals, making this combination more suitable for dynamic tracking than for rigorous quantitative analysis [43]. These dye-dependent trade-offs also reflect broader methodological limitations inherent to lipid-mixing FRET assays.

FRET-based lipid mixing assays are widely used in membrane fusion research, but their capacity for rigorous quantification is limited. Calibration curves are generally derived from homogeneous membrane systems and do not adequately reflect the heterogeneity in vesicle size or the localized nature of lipid mixing during actual fusion events, which can introduce systematic deviations in quantitative estimates [44,45]. In addition, fluorophores are susceptible to photobleaching, and FRET readouts are strongly influenced by probe distribution and aggregation within the membrane; variables unrelated to fusion may therefore confound signal interpretation [46]. FRET efficiency is also sensitive to membrane curvature, such that highly curved vesicles or dynamic membrane deformations can reduce quantitative reliability [45]. Moreover, non-fusogenic vesicle adhesion may generate spurious FRET signals that are difficult to correct for experimentally [45]. Taken together, these factors limit the use of lipid FRET assays for absolute quantification and make them better suited to semi-quantitative analysis and the characterization of kinetic trends.

Even with these limitations, lipid-mixing FRET assays remain useful in many experimental contexts. By converting nanoscale membrane reorganization into measurable changes in fluorescence ratios, they offer a practical and sensitive approach for in vitro studies of SNARE-mediated membrane fusion. Such assays are particularly well suited to kinetic measurements, small-scale screening, and exploratory mechanistic studies, providing a functional link between molecular-level interactions and observable fusion outcomes.

### 2.2. Content Mixing-Based Detection of Membrane Fusion

The field of membrane fusion research has traditionally relied on a core sensing paradigm that assumes a direct and necessary spatiotemporal coupling between the lipid bilayers mixing and the contents mixing. Guided by this assumption, FRET sensors based on lipid mixing have become classic detection tools, thanks to their simple construction and intuitive signal response [18]. Nevertheless, the reliability of this idea is now being doubted. Recent evidence suggests significant decoupling between lipid mixing and content exchange. In some DNA-mediated membrane fusion systems, for example, FRET sensors based on lipid mixing report up to 80% efficiency. In contrast, sensors that track content exchange, including those using labeled DNA strands, indicate content mixing rates below 2% or even no mixing at all [11,47]. Similar results have been seen in fusion driven by polyethylene glycol [48].

These findings show that lipid mixing signals alone are not enough to prove that a fusion pore has opened or that fusion is complete. To directly detect fusion pore formation and content exchange, researchers developed fluorescence sensors based on content dilution. An early representative approach exploits the concentration-dependent self -quenching of small-molecule dyes, such as carboxyfluorescein, calcein, and sulforhodamine B. These dyes are quenched when encapsulated at high concentrations within vesicles; however, once fusion triggers their release and dilution, the fluorescence intensity will recover significantly [49,50,51].

**Figure 3:**
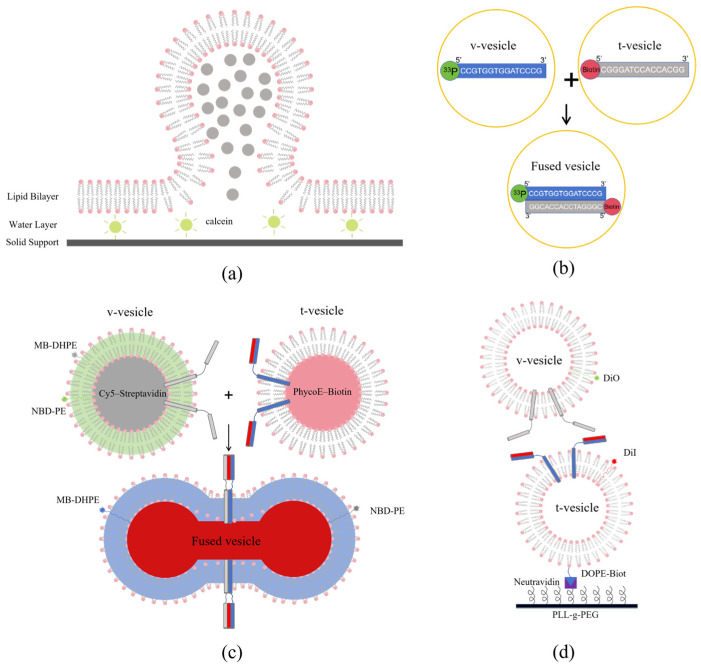
Diverse implementation pathways of membrane fusion sensing technologies. (**a**) Schematic diagram of the content mixing sensor for detecting membrane fusion. High-concentration calcein (grey) undergoes self-quenching when encapsulated inside vesicles. After fusion with the supported lipid bilayer, the internal calcein is released into the water layer with a larger volume. The resultant dilution relieves self-quenching, leading to fluorescence recovery (depicted as green). (**b**) Schematic diagram of the oligonucleotide-based membrane fusion assay. Content mixing between v-vesicles and t-vesicles is detected via a complementary oligonucleotide pair: one strand is 5′-^33^P-labeled (in v-vesicles), and the other is 5′-biotinylated (in t-vesicles). After vesicle fusion and detergent-mediated lysis, the biotinylated DNA is captured by immobilized streptavidin, and fusion events are quantified by measuring the associated ^33^P radioactivity. (**c**) Schematic representation of complementary FRET-based assays for simultaneous monitoring of lipid and content mixing during membrane fusion. Lipid mixing is tracked via dilution-dependent decrease in FRET between membrane-embedded NBD-PE (green donor) and MB-DHPE (blue acceptor), resulting in an emission shift from green to blue. Content mixing is reported by affinity-driven FRET increase: upon streptavidin-biotin binding, Cy5-labeled streptavidin (far-red donor) and phycoerythrin-labeled biotin (orange-red acceptor) come into proximity, elevating FRET and shifting emission from orange-red to far-red. (**d**) Single vesicle fusion assay based on surface-immobilized t-vesicles. t-vesicles are immobilized onto a PEG-coated glass surface via biotin (blue)-neutravidin (purple) coupling. Fusion of individual v-vesicles with t-vesicles is monitored by fluorescence microscopy. DiO (green) and DiI (red) are reconstituted into v-vesicles and t-vesicles membranes, respectively. Vesicle fusion reduces the distance between dyes, leading to increased FRET efficiency. Note: image not drawn to scale.

Among these, carboxyfluorescein was the first to be applied, establishing the methodological foundation for the field [50]. In contrast, calcein offers superior stability, as it is less susceptible to the pH changes and oxidation that affect carboxyfluorescein [52]. By leveraging calcein’s quenching properties, researchers validated that the interaction between syntaxin and synaptobrevin is both necessary and sufficient for vesicle docking and fusion (Figure 3a) [49]. Calcein has also expanded its utility through its capability for chelating metal ions [53]. Meanwhile, sulforhodamine B provides unique advantages for dynamic membrane studies; its red fluorescence avoids spectral crosstalk with common green probes, facilitating multicolor labeling with minimal membrane disruption. Such studies revealed that in SNARE-mediated fusion, lipid mixing and pore opening occur almost synchronously [54].

Despite their utility, when the small-molecule dye sensors are applied to SNARE protein systems reconstituted at physiological ratios, their inherent limitations become evident. Subtle disturbances in membrane structure readily induce nonspecific probe leakage, resulting in substantial background noise that severely compromises the specificity and reliability of the sensing system [55,56,57].

To develop sensors with lower background and higher specificity, the Rothman team innovatively designed a sensor based on DNA hybridization. Complementary DNA strands labeled with ^33^P and biotin were separately encapsulated within two distinct vesicle populations. Only upon membrane fusion, forming a continuous lumen, could the DNA strands encounter and hybridize, subsequently enabling capture by streptavidin-coated magnetic beads and generating a radioactive signal (Figure 3b). This strategy effectively transforms content mixing into a specific molecular binding event [58].

The two systems differ substantially in their quantitative sensitivity and robustness, particularly with respect to the limit of detection (LOD), signal response characteristics, and resistance to interference. The small-molecule dye dilution assay typically exhibits a quantitative LOD of ≥5% fusion, limited by incomplete dye self-quenching and background noise arising from membrane leakage [59,60]. At lower fusion efficiencies (<5%), weak fluorescence changes cannot be reliably resolved, and background fluctuations further exacerbate quantitative deviations [61]. In contrast, the DNA hybridization system benefits from minimal background signal and intrinsic signal amplification, achieving an ultralow LOD of <1% fusion without a pronounced response threshold. Single-molecule hybridization events can therefore generate detectable signals even under conditions of limited content mixing, supporting stable quantitative analysis in low-efficiency fusion systems (Table 1) [62,63].

Liu et al. also introduced a multi-channel fluorescence sensing system. This system integrates lipid mixing sensing (using Marina Blue PE and NBD PE FRET pairs) with content mixing sensing (using PhycoE biotin and Cy5 streptavidin FRET pairs) [21]. This design enables dual verification and synchronous monitoring of actual fusion events at the population level (Figure 3c) [21]. By introducing specific biomolecular interactions as molecular switches, these sensors significantly enhance the signal-to-noise ratio and detection reliability.

Since these sensors represent ensemble averages, they cannot distinguish intermediate states such as docking, hemifusion, and complete fusion. A further limitation is their inability to correlate individual fusion events with protein conformational changes. Therefore, developing next-generation technologies with real-time, single-vesicle, and multiparameter resolution is necessary.

### 2.3. Evolution of Optical Sensing Technologies for Membrane Fusion

To overcome these limitations and achieve real-time observation of single events with high spatiotemporal resolution, membrane fusion research has entered a new phase of single vesicle sensing. The essence of this approach lies in integrating spectrally distinct fluorescent probes with advanced microscopy imaging technologies, thereby creating precise sensing systems that enable simultaneous multiparameter monitoring of individual vesicles. In practice, lipid components can be labeled with ATTO 465 DOPE (excited at 488 nm), while content can be encapsulated with SRB (excited at 532 nm). Alternatively, a combination such as ATTO 647N-DOPE (647 nm excitation) paired with Alexa Fluor 488 (488 nm excitation) can be employed. The selection of these spectral pairs enables complete signal separation in multicolor imaging [51].

Confocal microscopy sensing platforms serve as foundational instruments for these investigations. By leveraging optical sectioning and precise localization, researchers can manipulate and monitor individual giant unilamellar vesicles (GUVs) across multiple spectral channels. In one representative study, researchers delivered large unilamellar vesicles (LUVs) near a targeted GUV via microinjection. This configuration allowed for the simultaneous tracking of lipid mixing efficiency (using DPPE NBD and DPPE Rh FRET pairs) and content mixing (via Atto dye intensity shifts), while also recording the morphological fluctuations of the membrane. Such integrated measurements facilitate a multidimensional and quantitative characterization of fusion events [45]. Another study used DiO and DiI FRET pairs to differentially label v-vesicles and t-vesicles. By exploiting the binding between DOPE Biotin and neutravidin, researchers immobilized the t-vesicles on a PEG-modified glass surface. The interactions between freely diffusing v-vesicles and t-vesicles are monitored in real time using confocal microscopy. This sensing system not only clearly distinguishes between a FRET-negative “docked” state and a high-FRET “full lipid-mixing” state but also captures a low-FRET intermediate state indicative of “partial mixing”. These observations provide direct evidence for the existence of hemifusion intermediates and reveal the diversity of vesicle fusion pathways (Figure 3d) [22].

Compared to confocal microscopy, TIRF microscopy offers unmatched surface imaging signal-to-noise ratios and high-speed imaging capabilities. These features are critical for resolving individual fusion events at a millisecond scale. TIRF utilizes an evanescent field that only excites fluorescent molecules within approximately one hundred nanometers of the glass surface. This selective excitation results in extremely high signal-to-noise ratios, making it ideal for monitoring rapid interactions between vesicles and planar target membranes [25].

One study applies TIRF to observe the de-quenching of R18 dyes as a marker for the docking and fusion of small unilamellar vesicles (SUVs) with planar lipid bilayers. This approach revealed how fusion events are influenced by vesicle size, providing a novel methodology for studying peptide-mediated membrane fusion and designing drug delivery systems [64]. And another research using polarized TIRF and fluorescent lipids such as LR-PE tracked the docking, fusion, and lipid transfer kinetics between vSUVs and tSBLs. These experiments found that fusion pore flickering limits lipid release. The data also indicated that the degree of pore opening correlates weakly with vesicle area and v-SNARE copy numbers. These insights provide a new perspective on the kinetic mechanisms of membrane fusion pores [26].

Going beyond basic intensity imaging, sensing techniques based on advanced spectroscopy offer deeper molecular insights. Two-photon fluorescence cross-correlation spectroscopy (FCCS) excels at capturing dynamic interaction processes. By analyzing the synchronization of dual color fluorescence intensity fluctuations, FCCS provides a non-invasive, in situ report of the initial binding kinetics and affinity between vesicles and target membranes. This capability makes it an exceptional sensor for identifying specific docking events [65,66]. Researchers have utilized dual color labeling (using dyes such as Texas Red and Oregon Green) to accurately distinguish between free, docked, hemifusion, and full fusion states. This approach enables detection of membrane proximity within the 10-nanometer range and is adaptable to various experimental conditions, including different lipid compositions and protein combinations. These studies have provided important experimental evidence and mechanistic insights into the molecular mechanisms of protein-mediated membrane interactions, particularly during synapse-related membrane fusion [66].

Time Correlated Single Photon Counting (TCSPC) has become an important molecular-level tool in membrane fusion research. When FRET occurs between a donor and an acceptor, the fluorescence lifetime of the donor decreases measurably. By quantifying this lifetime change, FRET efficiency can be determined more directly and with reduced influence from spectral crosstalk or variations in probe concentration. For these reasons, TCSPC is widely regarded as a highly reliable approach for quantitative FRET measurements [67,68]. TCSPC is particularly well suited to single-vesicle systems, which are often characterized by low photon counts, weak fluorescence signals, and rapid dynamics. Because lifetime measurements are generally less dependent on excitation intensity, fluorophore concentration, photobleaching, and detection efficiency than intensity-based readouts, TCSPC typically provides improved quantitative stability and resolution compared with methods that rely solely on fluorescence intensity [69,70]. However, TCSPC also presents practical challenges, including high instrumentation costs, relatively long acquisition times, complex data analysis, and stringent experimental requirements. In applications such as high-throughput analysis, rapid screening, or routine qualitative observations, intensity-based FRET approaches may offer greater practicality due to their simpler implementation and faster imaging speeds [71,72]. Accordingly, the strengths of TCSPC are most evident in high-precision, quantitative investigations at the single-vesicle level, rather than as a universal solution for all FRET-based experiments.

The practical utility of this method is demonstrated by studies where NBD-PE and Rh-PE served as a FRET donor-acceptor pair to label single vesicles. Through the use of TCSPC to monitor individual fluorescence lifetime shifts, researchers achieved a precise quantification of single vesicle fusion. By avoiding the averaging effects inherent to bulk measurements, this work confirmed that lipid composition and vesicle size are critical determinants of fusion dynamics [68].

Membrane fusion research has fundamentally shifted from a paradigm of simple lipid mixing toward an era of integrated sensing characterized by multi-parameter, real-time, and single-event detection. The synergy between lipid mixing sensors and content mixing sensors provides essential cross-validation. This integrated framework reveals the complex temporal relationship between lipid reorganization and content release while establishing a more rigorous and complete detection methodology. Confocal and TIRF imaging platforms enable the visualization of the fusion process, while spectroscopic sensors such as TCSPC and FCCS provide the capability to quantitatively resolve molecular states and interactions.

The future development of sensing technologies will continue to refine our understanding of the precise molecular landscape of membrane fusion. Advances in probe design, particularly those offering higher spatiotemporal resolution and lower background noise, remain pivotal. Equally important will be the integration of optical sensors with other physical detection methods, providing indispensable tools for uncovering the remaining mysteries of biological membrane dynamics.

## 3. Principles and Applications of Electrochemical Sensors

### 3.1. ND-BLM-Based Sensors

BLM is a classic artificial planar lipid bilayer model. Since its introduction in 1962, the BLM has opened a window for studying membrane functions due to its structure and electrical characteristics that closely resemble those of natural cell membranes. This model is particularly effective for characterizing the electrical properties of bare bilayers [73]. Nanodiscs are prepared by wrapping two copies of a membrane scaffold protein (MSP) around a lipid bilayer [74]. These structures offer significant advantages for biosensing, including ease of preparation, high size uniformity, and precise control over the number of surface proteins. As the nanodisc is at the nanoscale, the fusion pore is also nanoscale and unable to expand [75]. This effectively captures the fusion pore in its initial open state, providing a foundation for studying its kinetic properties. By exploiting the electrophysiological adaptability of BLM and the structural advantages of nanodiscs, researchers integrated them to construct a high-sensitivity ND-BLM electrophysiological sensing system. This system was employed to monitor pivotal events in membrane fusion.

As the fundamental unit of sensing, the formation of BLM and verification of its integrity are essential. Briefly, the procedure utilizes artificial lipids, such as 1,2-diphytanoyl-sn-glycero-3-phosphocholine (DphPC), 1,2-dioleoyl-sn-glycero-3-phospho-L-serine (DOPS), and 1,2-dioleoyl-sn-glycero-3-phosphoethanolamine (DOPE), among others, which are assembled into planar bilayers across small apertures separating two chambers via the painting or folding method to simulate the physical environment of cell membranes [73,76].

Although BLM systems are widely used in membrane fusion studies because of their excellent temporal resolution, reproducibility remains a practical challenge. Bilayer formation often relies on manual film painting, and small variations in coating position, lipid distribution, or aperture material can lead to preparation-to-preparation differences. As a result, membrane capacitance is typically controlled within a defined range rather than maintained at a single fixed value, reflecting unavoidable variations in bilayer thickness and geometry [77]. The sealing quality of the bilayer determines the seal resistance, which strongly influences the noise characteristics of the recording. Higher and more stable seal resistance generally corresponds to lower background noise and improved signal-to-noise ratio, underscoring the importance of careful control during bilayer formation [78].

**Figure 4:**
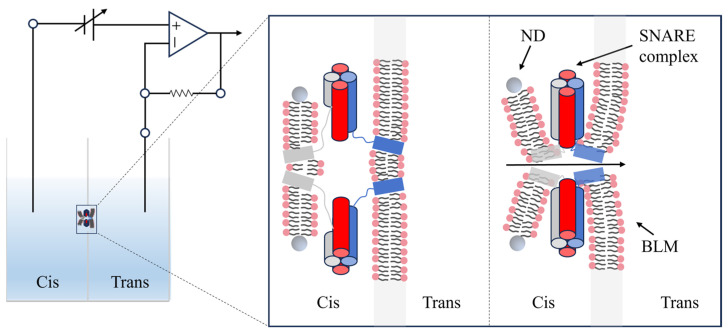
Schematic drawing of the nanodiscs-black lipid membrane (ND-BLM) system. The experimental principle is shown in the left panel. The middle and right panels show the process of SNARE complex formation and the opening of the fusion pore. SNARE proteins are depicted as rod-like structures on the membranes: blue and red denote t-SNAREs, and grey denotes v-SNARE. The gray circle represents the MSP protein. Note: image not drawn to scale.

Further noise reduction can be achieved by selecting low-noise, high-sensitivity, wide-bandwidth amplifiers (such as low-noise voltage-clamp amplifiers or transimpedance configurations) (Figure 4), optimizing circuit parameters, and reducing aperture size [79]. A smaller aperture not only limits capacitive and leakage noise but also enhances mechanical stability, thereby extending bilayer lifetime and enabling reliable recording of picoampere-level single-channel currents [80].

To assess the quality and stability of the membrane, small triangular voltage pulses are applied to the bilayer, and the resulting current response is recorded in real time [81]. The membrane capacitance is subsequently derived from the current–voltage relationship (C = I/(dV/dt)), where I is the measured current, and dV/dt represents the rate of change in voltage with respect to time, enabling evaluation of the bilayer integrity and stability.

In practical applications, t-SNARE anchors to the BLM via vesicles. It then binds to nanodiscs that carry v-SNARE and other membrane proteins, such as synaptotagmin-1, forming SNARE complexes that trigger the opening of fusion pores. Once a fusion pore opens, ions flow across the membrane driven by potential or concentration gradients, generating an electrical current. This design turns the complex process of membrane fusion into simple, measurable electrical signals.

This sensing system has shown significant potential in studying the molecular mechanisms of membrane fusion. By analyzing parameters such as conductance fluctuations and I-V curves, the system can track how fusion pores form and evolve. And this also allows researchers to quantitatively measure how membrane proteins and lipids affect pore size and stability.

**Figure 5:**
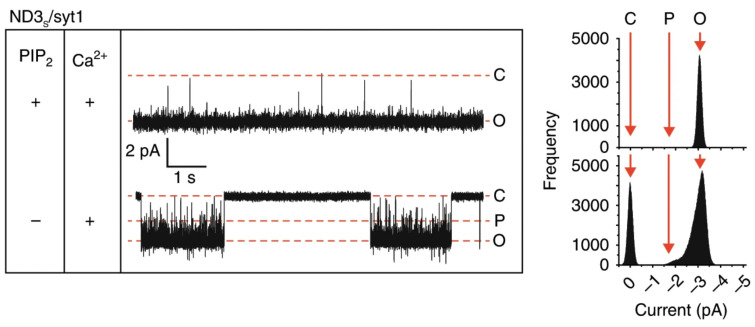
Traces of single fusion pores, formed using ND3S/syt1 plus Ca^2+^, with (+, upper panel) and without (−, lower panel) 2% PIP2 in the BLM. Respective current histograms are shown beside the traces. Closed (C), open (O), and partially open (P) states are shown. Reprinted from Ref. [28].

Through fusion pore “flicker” behavior, conductance fluctuations, and competitive inhibition experiments, Bao et al. proved the dynamic reversibility of trans-SNARE complexes [27]. With sub-millisecond temporal resolution, Das et al. tracked the kinetic trajectory of fusion tripped by Ca^2+^ and Syt1 (Figure 5) [28]. Their work clearly showed how multiple intermediates shift to synchronized initiation. By observing sub-conductance states, they also revealed how the NSF molecular machine assembles and disassembles. This system is also highly flexible in controlling the lipid environment. Although fusion pores are believed to be jointly formed by SNAREs transmembrane domains (TMDs) and lipids, the exact role of lipids still needs more studies [29]. Wu et al. used this system to precisely adjust cholesterol levels in the lipid bilayer to see how it affects fusion pore dynamics [30]. Through the analysis of the duration of fusion pore open and closed states, the team proved that cholesterol stabilizes the open state by changing membrane bending rigidity [30]. Compared to traditional cell studies, this platform without off-target effects and allows real-time control of cholesterol levels.

The integration of nanodiscs and BLM offers this system several advantages. The sub-millisecond temporal resolution ensures the precise capture of transient events during membrane fusion, providing critical support for resolving rapid molecular dynamics. As in vitro models, both nanodiscs and BLM offer high controllability and low interference. They remove the noise from complex intracellular environments and allow for the precise adjustment of membrane components and receptors [75,82]. These features enable membrane fusion research to reach the single-molecule level, establishing the system as a central tool for understanding membrane proteins’ function and pore kinetics.

### 3.2. SLB-Based Sensors

While sensing technologies based on ND-BLM have demonstrated outstanding performance in membrane fusion studies, they are often fragile and typically last only a few hours. This instability makes it difficult to use for long-term monitoring or to combine with other methods [83]. This situation changed completely with the development of SLBs.

Due to its substrate support, SLB is highly stable and reproducible. It can also work with surface-sensitive characterization techniques, including electrochemical impedance spectroscopy (EIS), surface plasmon resonance (SPR), and quartz crystal microbalance with dissipation (QCM-D) [36]. It has evolved into an advanced platform for both membrane fusion mechanism research and biosensing applications.

The amphiphilic nature of lipid molecules enables the construction of SLB into continuous and uniform bilayer structures on substrate surfaces through classical self-assembly techniques. These include Langmuir–Blodgett (LB) film transfer, Schaefer dipping, and vesicle fusion [37,38]. The lipid bilayer is stably anchored to a solid substrate through a combination of Van der Waals forces, electrostatic interactions, hydration forces, and steric effects.

**Figure 6:**
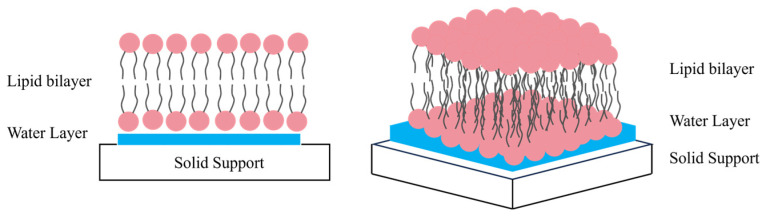
Schematic illustration of a solid-supported bilayer. Note: image not drawn to scale.

Between the membrane and the substrate, a thin water layer with a thickness of approximately 1 to 2 nm is formed. This water layer acts as a critical lubricant, preserving the lateral mobility of both lipid molecules and embedded proteins within the membrane. Such a structural arrangement ensures the maximum maintenance of membrane integrity and biological functional activity (Figure 6).

When SLB is constructed on conductive substrates such as indium tin oxide (ITO), gold, or doped semiconductors, its physicochemical properties can be monitored in situ through electrochemical methods [39]. Electrochemical impedance spectroscopy (EIS) has emerged as a powerful, non-invasive, and label-free sensing technique with high sensitivity for evaluating biorecognition events occurring at the membrane interface [40].

This sensing technology operates by applying a broadband, low-amplitude alternating voltage in the millivolt range across the detection system and recording the corresponding current response. From these measurements, the impedance of the electrode–membrane–electrolyte system is determined. These data are then fitted to an equivalent circuit model to extract critical electrical parameters, including the resistance and capacitance of the lipid bilayer. In SLB-based fusion assays, impedance spectra are typically analyzed using a simplified parallel resistor–capacitor (RC) model, since faradaic charge-transfer reactions are generally minimal [84]. This approach enables quantitative evaluation of membrane restructuring during fusion through changes in these electrical parameters.

In early SLB-based electrochemical sensing systems, gold served as the most widely used conductive substrate due to its excellent electrical conductivity. However, it exhibits significant limitations as a high-performance sensor. On one hand, the interfacial impedance of gold electrodes is relatively high, which directly restricts the detection sensitivity and the lower detection limit of the sensing system. On the other hand, the gold surface is naturally hydrophobic, often requiring complex chemical modifications to induce SLB formation. This requirement increases both the operational complexity and the uncertainty of experimental outcomes [85,86].

The emergence of poly(3,4ethylenedioxythiophene): poly (styrenesulfonate) (PEDOT:PSS) provides an effective solution to the aforementioned limitations. PEDOT:PSS is a hydrophilic polymeric film that supports the formation of unique polymer-supported lipid bilayers [87]. This material benefits from high electrical conductivity and ion permeability. These features expand the effective electrochemical surface area, leading to a significant reduction in system impedance. This reduction improves the sensitivity to weak signals generated by molecular interactions at the SLB interface [87,88]. PEDOT:PSS is highly stable and biocompatible under physiological conditions. SLB formed on its surface maintains high lateral mobility for both lipids and proteins. These features make PEDOT:PSS an ideal material for detecting subtle membrane-associated processes [87,89,90].

Recent studies have reported the development of a novel sensing platform by constructing SLB with natural membrane components on PEDOT:PSS electrodes. This platform effectively simulates the infection environment of enveloped viruses, such as SARS-CoV-2, and enables the quantitative characterization of infection progression through electrochemical signals.

**Figure 7:**
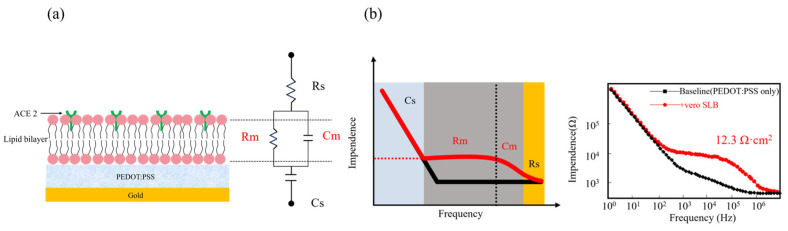
EIS was used to characterize the electrical properties of an SLB on a PEDOT:PSS electrode. (**a**) Schematic of the SLB assembly on a PEDOT:PSS modified electrode, an SLB is modeled electrically as a capacitor and a resistor connected in parallel. Note: image not drawn to scale. (**b**) Representative EIS data, its resistance Rm can be extracted by fitting to the RC circuit as shown. It can then be normalized by the area of the electrode. The black curve represents the baseline signal of the bare electrode (PEDOT:PSS only), while the red curve shows the circuit response upon self-assembly of a SLB on the electrode surface (+Vero SLB). Adapted from Ref. [91].

EIS measurements show that the bare electrode exhibits a “hockey stick” baseline, which represents a frequency-dependent impedance curve of a series resistance-capacitance (RC) structure. Following the self-assembly of the SLB on the electrode surface, the circuit response transitions to a “chair-shaped” profile. This change confirms the integration of parallel RC components, a well-established electrical characteristic of lipid bilayers. And equivalent circuit fitting allows for the precise extraction of the membrane resistance (Rm) and capacitance (Cm) of the SLB (Figure 7).

Research indicates that this platform can accurately quantify and distinguish viral binding/fusion events, entry pathways, strain-specific behaviors, and drug inhibition effects based on changes in membrane resistance [91]. Compared with traditional live cell infection assays, this in vitro sensing platform features a label-free electrical approach to monitor viral interactions with host cell membranes. It delivers rapid responses and short detection cycles through a simple operation. The system’s high reproducibility supports both the investigation of viral fusion mechanisms and the high-throughput screening of antiviral drugs.

Building on this technological framework, PEDOT:PSS can be scaled for integrated, high-throughput applications. Using microfabrication, the material is patterned into organic microelectrode array (OMEA) chips and functionalized with SLB. The integration of microfluidic technology with this setup enables automated fluid handling and parallel data collection [92], which significantly expands the platform’s practical utility and translational potential in biosensing.

In addition to being applied in EIS measurements, PEDOT:PSS is also used in OECTs [41]. As three-terminal sensing devices, OECTs feature a PEDOT:PSS conducting channel situated between the source (S) and drain (D) electrodes. The direct contact of the source, drain, gate, and channel with the electrolyte ensures a rapid response to variations in membrane properties. The sensing mechanism relies on gate-voltage-induced ion injection from the electrolyte into the channel, which modulates conductance by altering the doping state. The unique ion-to-electron coupling and subsequent amplification effect in OECTs convert subtle ionic fluctuations into significant current changes [42].

**Figure 8:**
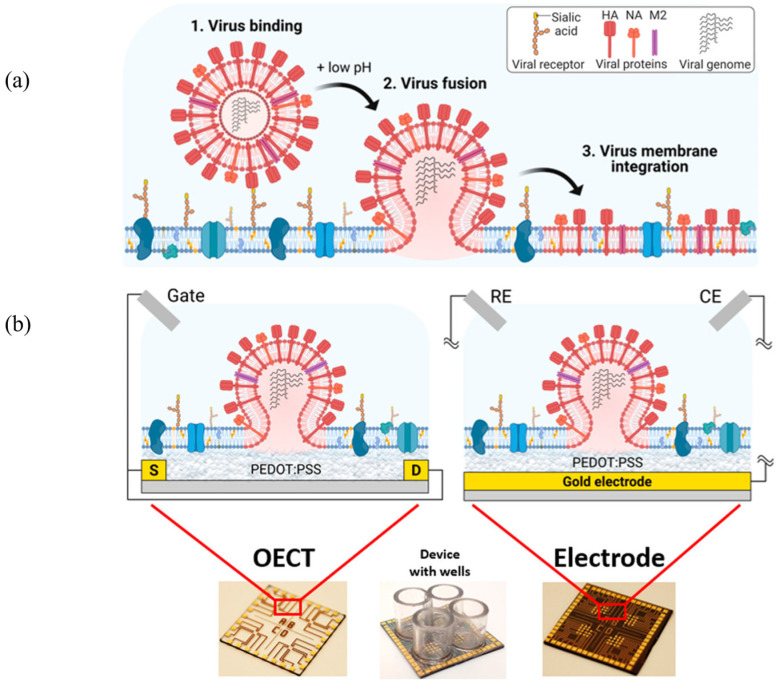
Diagram of the proposed sensing strategy. (**a**) Cartoon representation of enveloped viral entry processes, using influenza as the prototypical example. Influenza binds to a sialic acid receptor (yellow) on the host cell membrane, and the influenza hemagglutinin (HA) proteins are triggered to induce viral–host cell membrane fusion upon exposure to low pH. During this fusion event, the viral envelope lipids and proteins (red) mix with the host cell membrane (blue), allowing the virus to release its genome. Note: image not drawn to scale. (**b**) Images of the electroactive platforms used in this study, including OECTs (**left**) and MEAs (**right**). For scale, each device measures 2 cm × 2 cm. The OECT arrays, fabricated on glass, contain four distinct working areas that can be isolated with glass wells attached to the surface, as shown in the middle image. Each working area contains 4 OECT channels, each 50 μm in length and 50 μm in width, covered with PEDOT:PSS. The MEAs were fabricated on Si/SiO_2_ glass with a similar design. Each of the four working areas contains 15 round electrodes, each with a diameter of 500 μm. The inset shows a cartoon of the OECT and electrode sensing mechanism. The OECT consists of a conductive polymer (CP) channel located between the source (S) and drain (D) electrodes, which are in contact with an electrolyte and a gate electrode. The electrode has a CP layer formed on top of it and is configured with a reference electrode (RE) and a counter electrode (CE), all in direct contact with the electrolyte. In both setups, the biomimetic membrane is formed on top of the CP to probe virus interactions. Reprinted with permission from Ref. [41]. Copyright 2021 American Chemical Society.

In viral membrane fusion detection, adding an insulating SLB between the channel and gate changes the transient response time needed for channel current stabilization after applying a gate voltage pulse. As a result, the fusion state between viruses and host cell membranes can be monitored by calculating this response time. Research by Tang et al. using the influenza virus as a model system demonstrated that both EIS and OECTs-based measurements were employed to simulate the fusion of enveloped viruses with host cell membranes [41]. The results showed that, compared to the conventional EIS, the OECTs-based measurement method generally exhibits higher sensitivity in detecting the fusion events (Figure 8) [41].

SLB-based sensors have been widely adopted in viral fusion research. In these systems, substrate reinforcement provides mechanical robustness and facilitates optical accessibility. The planar geometry of SLBs also enables controlled receptor reconstitution and supports high-throughput measurements of viral binding and membrane fusion. These features make SLB platforms well suited for probing viral entry mechanisms and for comparative evaluation of viral variants. However, inherent structural constraints introduce certain limitations. The nanoscale sub-membrane reservoir beneath supported bilayers is susceptible to ion saturation or depletion [93]. These ionic imbalances can induce localized polarization and distort transmembrane potentials. Such electrochemical instability may compromise sustained monitoring of fusion pore conductance and reduce the quantitative resolution of late-stage pore dynamics. In contrast, ND-BLM-based platforms lack solid support and therefore avoid substrate-associated constraints. This configuration can be advantageous for prolonged electrophysiological analysis of fusion pore kinetics. Rather than functioning as competing systems, SLB and free-standing bilayer platforms address distinct experimental objectives. SLB systems are well suited to studies of viral entry mechanisms and population-level measurements, whereas free-standing bilayers are more appropriate for high-resolution electrophysiological analysis of fusion pore dynamics.

## 4. Summary and Outlook

Membrane fusion serves as a fundamental step in various biological functions. Among these, SNARE-mediated membrane fusion and viral entry into host cells are intrinsically linked to the pathological process. Although these two types of fusion differ in physiological function, they share a common molecular foundation where fusion proteins drive the local rearrangement of lipid bilayers to form fusion pores [94,95]. These mechanistic and kinetic parallels justify a joint discussion of the sensing technologies developed to monitor both processes.

In this review, we focus on electrochemical and optical sensing technologies within in vitro membrane fusion models. Distinct from in vivo systems, these reconstructed models minimize interference from the complex intracellular environment, allowing the fusion process to be resolved under controlled conditions. The simplified system also makes sensor integration, parameter adjustment, and quantitative signal analysis easier and more flexible. When forming lipid bilayers in vitro, it is crucial to recognize that lipid composition, membrane curvature, and the local membrane environment play significant regulatory roles in membrane fusion behavior [96]. Therefore, incorporating native biological membrane fragments into sensing platforms creates a more physiologically relevant state, helping to bridge the gap between in vitro models and authentic biological processes [89].

SNARE-mediated and viral fusion share molecular similarities, yet the measurement goals differ. SNARE-mediated fusion research typically focuses on pore formation, kinetics, and protein conformational changes. In contrast, detecting virus–host fusion requires high temporal resolution and sensitivity to capture rapid events and distinguish between viral strains. Thus, while sensing platforms generally have wide potential, their design must be tailored to the specific needs of membrane fusion instead of aiming for generic solutions.

In membrane fusion research, optical and electrochemical sensing each present specific strengths and limitations. Optical methods typically rely on fluorescent labels to visualize spatial details and conformational changes. However, these labels can introduce cytotoxicity and potentially interfere with the fusion process. Electrochemical sensing allows for label-free detection with high sensitivity and temporal resolution, but its data interpretation relies on model fitting, where the choice of methods can bias the results (Table 2). Thus, for complex scenarios, relying on a single sensing modality is often insufficient for a complete analysis.

Given these challenges, multimodal sensing approaches are increasingly critical. In the combined electrical and optical system, electrical signals monitor the formation and stability of fusion pores. Meanwhile, optical signals provide independent verification of content release and protein conformation, effectively minimizing false positives.

Recent studies have combined current recording with optical techniques, such as fluorescence recovery after photobleaching (FRAP) and single-particle tracking (SPT), using suspended membranes in microfluidic channels. Even though signal stability and accuracy in these integrated systems may fall short of standalone counterparts, the multimodal strategy offers valuable insights for developing future sensing platforms [105].

Membrane fusion sensing has evolved from qualitative observation to quantitative analysis. Using advanced platforms, researchers can now quantitatively characterize core fusion parameters. This capability provides objective evidence to distinguish between normal and mutant SNARE states, as well as different viral fusion states, while also establishing a foundation for drug screening. Recently, White et al. reported a CMOS-integrated electrochemical sensor that enables real-time monitoring of neurotransmitter release at the single-vesicle level [106]. This technology enables tracking treatments for psychiatric disorders, such as depression, offering direct insights into drug efficacy [106]. Such breakthroughs underscore the potential of fusion sensing to advance translational medicine.

Looking ahead, membrane fusion sensing continues to face several engineering challenges. Several areas merit further consideration as the field advances. One important aspect concerns the refinement of biomimetic design. In vitro sensing interfaces derived from natural vesicles may offer distinct advantages, as they more closely resemble the native microenvironment of membrane fusion and may enhance the physiological relevance of detection outcomes. Another area that warrants attention is electrode material optimization. Although simultaneous optical and electrochemical monitoring has become technically feasible, combined measurements often exhibit lower precision than standalone modalities. The development of integrated material platforms that exhibit both optical transparency and high electrical conductivity may alleviate compatibility constraints and improve the reliability of concurrent measurements. In addition, despite increasing device integration, automated analytical workflows remain relatively underdeveloped. Many platforms still require manual intervention for parameter adjustment. Incorporating autonomous optimization algorithms may enable more scalable and reproducible analytical modules, which could prove particularly valuable for high-throughput applications such as viral variant screening and antiviral drug discovery.

Encouragingly, the field is clearly shifting from single-parameter detection toward multiparametric measurements. As these platforms mature, they will uncover fundamental mechanisms and enable more reliable disease intervention and drug development.

## Figures and Tables

**Table 1:** Differences in quantitative sensitivity between the small-molecule dye dilution assay and the DNA hybridization system.

Dimension of Quantitative Sensitivity	Small-Molecule Dye Dilution Assay	DNA Hybridization System
Limit of Detection (LOD)	≥5% fusion ratio	<1% fusion ratio
Signal Response Threshold	Exists a critical dilution threshold, weak of undetectable response at low fusion ratios.	No apparent response threshold; signals can arise from single-molecule hybridization.
Anti-interference Capability	Weak; membrane leakage and environmental perturbations increase background noise.	Strong; low background signal and reduced sensitivity to membrane-related interference.

**Table 2:** Comparison of representative membrane fusion sensing platforms.

Sensor Category	Representative Platform	Temporal Resolution Spatial Resolution	Advantages	Limitations	Applications
Optical Sensors	FRET	millisecond; ~1–10 nm [97,98].	Simple and intuitive; Quantitative kinetic analysis; Independent of vesicle size.	Significant phototoxicity; Lack of intermediate state resolution.	Kinetic modeling; Small-scale drug screening; Basic mechanism studies.
	Confocal/TIRF	millisecond; ~250–500 nm laterally, ~500 nm axially/ ~100–250 nm laterally; ~60–100 nm axially [99,100,101].	Distinction of intermediate states (docking/hemifusion).	Phototoxicity and photobleaching; High equipment cost.	Single fusion events visualization; Diversity of fusion paths.
	TCSPC/FCCS	picosecond/millisecond; No fixed value; depends on the imaging system [102,103].	Molecular state quantification; Strong anti-interference ability.	Stringent probe requirements; Complex setup and low throughput.	Protein conformational changes; Precise intermembrane distance.
Electrochemical Sensors	ND-BLM-Based Sensors	sub-millisecond; ~1–5 nm [30].	Non-destructive; Label-free; Single-molecule level detection; Capable of capturing transient dynamics of fusion pores.	Mechanical fragility (short lifespan); Difficulties in integration.	Fusion pore dynamics; Single-molecule mechanism.
	SLB-Based Sensors	sub-millisecond; ~100 nm [91,104].	Non-destructive; Label-free; highly stable; Compatibility with surface-sensitive characterization techniques; Easy integration.	Susceptibility to ion depletion/saturation.	Viral binding/fusion assays; Rapid antiviral drug screening.

## Data Availability

No new data were created or analyzed in this study. Data sharing is not applicable to this article.
